# Strengthening the immunization supply chain: A time-to-supply based approach to cold chain network optimization & extension in Madhya Pradesh

**DOI:** 10.1016/j.vaccine.2021.09.062

**Published:** 2021-10-29

**Authors:** Varun Srivastava, Manish Ratna, Arindam Ray, Santosh Shukla, Vipin Shrivastava, Nitin Kothari, Amandeep Gupta, Meenal Kukreja, Harkabir Singh Jandu

**Affiliations:** a Clinton Health Access Initiative, 40, Okhla Phase III, Delhi 110020, India; b Bill & Melinda Gates Foundation, 5th Floor, The Capital Court, Olof Palme Marg, Munirka, New Delhi 110067, India; c Directorate of Health Services, Government of Madhya Pradesh, NHM Bhavan, Link Road No.3, Patrakar Colony, Bhopal 462003, India

**Keywords:** Vaccine supply chain optimization, Cold chain network extension, Immunization supply chain strengthening, Health facility location allocation, Time-to-supply

## Abstract

Expansion of immunization coverage is dependent in part on delivering potent vaccines in an equitable and timely manner to immunization outreach session sites from Cold Chain Points (CCPs). When duration of travel between the last CCP and the session site (Time-to-Supply) is too long, three consequences may arise: decreased potency due to exposure to heat and freezing, beneficiary dropouts due to delayed session starts, and, increased operational costs for the Health Facility (HF) conducting the outreach sessions. Guided by the Government of India’s recommendation on cold chain point expansion to ensure that all session sites are within a maximum of 60 min from the last CCP, CHAI and the State Routine Immunization Cell in the state of Madhya Pradesh collaborated to pilot a novel approach to cold chain network optimization and expansion in eight districts of Madhya Pradesh. Opportunities for realignment of remote sub-health centers (SHCs) and corresponding session sites to alternative existing CCPs or to HFs which could be converted to new CCPs were identified, and proposed using a greedy adding algorithm-based optimization which relied on health facility level geo-location data. Health facility geo-coordinates were collected through tele-calling and site visits, and a Microsoft Excel based optimization tool was developed.

This exercise led to an estimated reduction in the number of remote SHCs falling beyond the permissible travel time from CCPs by 56.89 percent (132 remote sites), from 232 to 100. The 132 resolved sites include 73 sites realigned to existing CCPs, and 59 sites to be attached to 22 newly proposed CCPs. Both the network optimization approach and the institutional capacity built during this project will continue to be useful to India’s immunization program. The approach is replicable and may be leveraged by developing countries facing similar challenges due to geographical, institutional, and financial constraints.

## 1 Introduction

Globally, the Expanded Programs on Immunization (EPI) rely on well-functioning immunization supply chains (iSC) to make efficacious and potent vaccines available to children in an equitable, timely, and efficient manner. In the resource limited public health settings of developing countries such as India, improvements to the iSC are even more critical, especially in light of new vaccine introductions for COVID-19 and other planned vaccines for diseases such as malaria, typhoid, and cholera, which will place additional burden on the already constrained and fragile supply chains [1]. Recognizing the demand for increased cold chain capacities due to the ongoing COVID-19 vaccine campaigns globally, World Health Organization (WHO) has called upon countries to assess their cold chain inventory and procure additional storage capacity and engage with private sector to address short term gaps while enabling long-term strengthening of the iSC [2] .

World Health Organization (WHO) guidelines state that all EPI vaccines must be stored at 2 °C- 8 °C in a cold chain at district level facilities or below [3]. Immunization cold chain, as a component of the iSC, is critical in delivering temperature sensitive vaccines to beneficiaries, as both heat and freezing exposures can damage the safety and efficacy of vaccines [4]. Insufficient capacity in the cold chain can affect the immunization services by reducing vaccine potency, increasing the burden on healthcare workers to conduct more outreach sessions, delaying new vaccine introductions, and increasing the risk of vaccine stock outs and wastage [5]. Hence, in order to sustainably increase the Full Immunization Coverage (FIC) in countries facing the dual challenge of new vaccine introductions and population growth, additional cold chain capacity is required at all levels of the iSC, necessitating an increase in both number of units of Cold Chain Equipment (CCE) and Cold Chain Points (CCPs). Accordingly, India’s vaccine cold chain capacity was augmented to meet the requirements of the COVID-19 vaccination campaign—and on average more than 5 Mn doses were administered daily during Aug’21. However, if the COVID-19 vaccination campaign paves the way for other adult vaccinations to be delivered through routine programs [6], further increases in overall storage capacity may be necessitated. This requirement is exacerbated because the new vaccines need more cold chain space as compared to traditional vaccines in EPIs, and are more expensive [7]. Adding distribution centers may also decrease the last-mile delivery and overall distribution costs, while increasing other costs related to setup and operations [8].

Vaccine availability is a challenge in developing countries and is impacted in part by storage capacity issues, timeliness of vaccine delivery, and healthcare worker burden [9]. Only adding more stationary cold storage capacity (in the form of new CCE) to existing CCPs increases the overall burden on the supply chain, by worsening the transport bottlenecks [10]. Thus, as India began expanding its Universal Immunization Programme (UIP) to make vaccines such as IPV (Inactivated Polio Vaccine), RVV (Rotavirus Vaccine), and PCV (Pneumococcal Conjugate Vaccine) available throughout the country, it considered opening new CCPs in addition to increasing cold storage capacity at existing CCPs. In the Comprehensive Multi Year Plan for Immunization 2018–22, the Government of India’s Ministry of Health and Family Welfare (MoHFW) recommended that health facilities in districts be identified for conversion to CCPs, with the aim of reducing travel time to outreach sessions (hereinafter referred to as “Time-to-Supply” or “TTS”) to less than 60 min [11].

### 1.1 India’s immunization supply chain and the relevance of Time-to-Supply

**Fig. 1:**
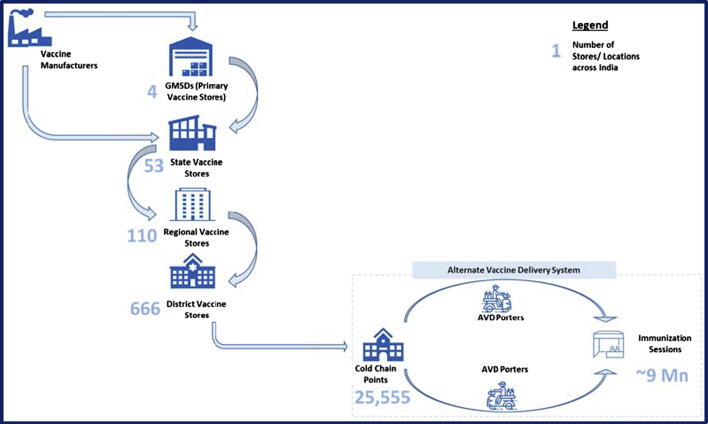
India’s immunization supply chain.

India’s iSC network consists of five tiers, with Government Medical Store Depots (GMSDs) at the top, followed by vaccine stores at the state, regional, district, and health facility (HF) levels, summarized in Fig. 1 [12]. The GMSDs receive vaccines from manufacturers and transport them to the state level stores, which in turn push the vaccines to regional or district stores. From the district stores, vaccines are transported to HF level CCPs, which are typically located at Community Healthcare Centers (CHCs) or Primary Healthcare Centers (PHCs). Additionally, around 150,000 sub-health centers (SHCs) serve India’s 700,000 villages, the lowest tier of public HF. At these SHCs, Auxiliary Nurse Midwives (ANMs) are present to provide basic healthcare support and to conduct immunization outreach sessions at Anganwadi Centers (AWCs) in nearby villages [13].

In order to ensure the timeliness of outreach sessions, vaccine safety and quality, and to reduce vaccine wastage, the MoHFW introduced the Alternative Vaccine Delivery System (AVDS). Under this system, the delivery of vaccine carriers containing vaccines, diluents, and conditioned ice packs from the CCP to the session sites is done by support staff comprising of porters (motorcycle and auto-rickshaw drivers etc.) [14].

Freezing during transportation is a widespread problem that impacts the cold chains of both developed and developing countries, including India [15], [16]. The adverse effect of freezing exposures on vaccine potency is permanent and irreversible [5], and the effects are aggravated due to agitation [17], which is likely during transportation, implying that multiple or prolonged exposures occurring during long transit times could be particularly impactful to vaccine potency. In India, greater travel times have been found to be positively associated with decreased vaccine potency [18].

Lack of well-conditioned ice packs is the main cause of freezing during transportation [19], [20]. The process of conditioning of ice packs is time consuming and may require between 90 and 120 min at an ambient temperature of 20◦C [21]. If the travel time to an immunization session site is too long, healthcare workers at CCPs may not be able to condition the ice packs adequately, as this may delay the start of immunization sessions. If the start of immunization sessions is delayed, wait times for beneficiaries may increase, which affects perceived quality of care and in turn negatively affects the decision to vaccinate [22]. Additionally, long distances to immunization session sites increase the operating costs for the HFs conducting the outreach sessions [23].

While the expected cool life of vaccine carriers with conditioned icepacks is expected to be greater than 30 h [20], this has not been studied during session conditions where, due to repeated opening and closing of the carriers by the health workers, the vaccines may be exposed to the external environment more often than in laboratory conditions. Additionally, the ice packs are removed from the vaccine carrier, and placed on the table during RI sessions [24]. In the Indian state of Madhya Pradesh, where temperatures frequently exceed 40◦C, this may mean that vaccines are exposed to non-optimal temperatures especially since immunization sessions are held for a duration of seven hours in India, starting at 9 AM [25].

Distances to outreach sessions from last CCPs are especially relevant in India, because of its Open Vial Policy (OVP) which covers seven out of the 12 vaccines in India’s UIP and mandates that open vials of DPT, TT, Hep-B, OPV, PCV, IPV and Pentavalent vaccines be returned to the CCPs following an outreach session [26]. The returned open vials may subsequently be used in a period of 28 days, as long as certain conditions relating to transportation, storage, and usage of the opened vial are met. Additional measures, such as keeping opened vials away from water and such as from thawing ice or defective ice packs need to be taken [27].

With these considerations in mind, CHAI and the State Routine Immunization Cell in Madhya Pradesh collaborated to optimize and extend the cold chain network in eight out of 52 districts in the state through a Time-to-Supply based optimization approach, while simultaneously considering the administrative, economic, and operational constraints. This paper describes the activities undertaken by the authors in the state for cold chain network optimization and expansion and the results obtained from it.

### 1.2 Literature review

The challenge of expanding the last tier of CCPs in India thus condensed to a location-allocation problem of optimally identifying store locations from existing HFs in order to set up new CCPs, while meeting the guideline related to Time-to-Supply. While this facility location-allocation problem has been considered in research in a variety of contexts, there is a conspicuous lack of academic research on the subject for vaccine distribution [28]. The focus of research on redesigning vaccine supply chains has thus far been on gaining operational efficiencies through the removal of one or more supply chain network layers, typically at district or regional levels [29], [30], [31]. Hirsch Bar Gai et al. expand on this by including the problem of location-allocation of the national and regional vaccine distribution centers in Nigeria [32].

While the research on iSC Network Design has not addressed the problem of location-allocation for the last tier, there are several studies that examine this issue more broadly in other types of HFs. Covering-based and median-based problems are two of the most common location-allocation problems for HFs. Covering-based problems seek to identify supply locations that are within a specified distance (or time) of demand locations, whereas median-based problems seek to minimize the total weighted distance costs between demand and supply locations [33]. Covering-based problems may be further classified as those seeking to minimize the number of supply or candidate locations while covering all demand points (Location Set Covering Problem, or LSCP), those seeking to maximize coverage of demand points given a fixed number of facilities which may be established (maximal coverage location problems, or MCLP), and those seeking to minimize a location dependent parameter such as distance or time while ensuring coverage of all demand points (p-center location problems) (see Table 1). The LSCP formulation requires that all demand points be covered by at least one facility within the given distance or time [34] but in case the demand points are sparsely located and have no supply point within the specified distance or time, this constraint may not be satisfied. On the other hand, MCLP formulation acknowledges that due to budgetary and programmatic constraints, complete coverage of demand points may not be possible. MCLP has been used for formulating the HF location-allocation problem in several studies, such as for determining outreach session locations for vaccination [35], for determining locations of community clinics in developing countries [36], and for location modelling of HFs in Malaysia [37].

**Table 1:** Types of Covering based location problems.

Type of problem	Objective	Constraint
Location Set Covering Problem (LSCP)	Minimization of number of supply facilities established	All demand points must be covered
Maximal Coverage Location Problem (MCLP)	Maximization of number of demand points covered	Maximum number of supply facilities that may be established is fixed
p-center	Minimization of maximum travel distance among all demand points and supply facilities	All demand point must be covered

## 2 Methodology

The location-allocation of expanding and optimizing the CCP network to cover session sites within 60 min time-to-supply was thus formulated as an MCLP problem. This was done with the objective of maximizing coverage of demand points (immunization session sites) while keeping the maximum number of CCPs that may be established limited to the total number of candidate locations available (in this case the HFs presently not a CCP). However, to justify opening a new CCP, a minimum level of utilization of the HF needs to be ensured due to the costs related to both setup and operations for the CCP. In this case, the hiring of an AVDS porter was found to be the major constraint since they are paid a fixed monetary incentive for each trip from CCP to the session site. Porter profitability, which is dependent on their fixed, operating, and opportunity costs, cannot be ensured unless a minimum number of SHCs and session sites are attached to a CCP. This constraint was added exogenously to the optimization problem described in subsequent sections, and was satisfied by identifying additional SHCs from surrounding areas of the CCP.

To calculate the minimum number of SHCs to be attached to each CCP, an economic breakeven analysis for the AVDS porters was carried out as a precursor to the network optimization and extension. Session sites are classified as “hard-to-reach” if they lie beyond 30 km from the associated CCP [38] and the compensation for trips to these session sites is higher than for “easy-to-reach” sites. All possible distributions of “hard-to-reach” and “easy-to-reach” sites were considered for evaluating the AVDS porter compensation scenarios. Costs related to capital expenditure on vehicle ownership, operating expenditure incurred by the porter on maintenance of the vehicle, fuel, insurance, and financing costs were considered to calculate the breakeven point (BEP). The BEP was computed as a function of profits through employment as a porter over and above the opportunity costs of alternative employment [39].

The research for the network optimization and extension was then divided into three phases. The first phase required identification of remote session sites with Time-to-Supply from CCPs greater than 60 min. In the second phase, realignment of session sites to existing alternative CCPs was explored, as this solution simply required an administrative rearrangement with no additional monetary investment at the CCP level. Following the results of the second phase, other HFs (which were presently not a CCP) were identified as candidate locations for opening new CCPs which would be responsible for delivering vaccines to the remaining session sites not meeting the Time-to-Supply guideline (with the constraint that each new CCP serve at least a minimum number of SHCs and their attached session sites, calculated through the AVDS porter economic analysis).

### 2.1 Data collection

For identification of remote session sites, geo-coordinates of all session sites in the eight districts were required. A survey tool was developed to capture the geo-coordinates and the facility details. A field force of volunteers was equipped with handheld smart devices to aid data collection efforts. For calculation of travel time between locations, Google Maps Distance Matrix API was used which calculated travel times based on motorable roads.

Capturing location information of all session sites proved to be challenging because of isolated and remote locations which made physical access arduous, internet connectivity issues which handicapped the data collection efforts, budget constraints, and inaccuracy in the collected data due to identically or similarly named villages. Hence, the travel time from the CCP to the SHCs was used as the proxy for session site locations since geo-coordinates information of SHCs could be captured with greater accuracy.

To obtain the existing administrative mapping of SHCs and associated CCPs, tele-callers were employed to collect the information by contacting all CCPs via phone. The tele-callers were able to collect data for 1464 SHCs, out of the 1500 SHCs present in the eight districts.

### 2.2 Step 1: Identification of remote sub health centers and associated session sites

Since the Time-to-Supply guideline of 60 min applied to session sites and not SHCs, optimization called for the time constraint to be adjusted to reflect the additional travel time from an SHC to a session site. Information on actual travel times was collected from healthcare staff for a randomly selected sample of SHCs. It was found that for the average case it took 20.12 min of travel from a SHC to a session site; the Time-to-Supply guideline for the purpose of optimization was reduced to 40 min to reflect this. Additionally, it was observed through validation with the program managers that Google Maps District Matrix API overestimated travel times, with an average overestimation of about 30 percent. Thus, a downward correction factor of 30 percent was applied to the travel times obtained from the service.

All SHCs for which the motorable travel times (Time-to-Supply) from associated CCPs was greater than 40 min were classified as remote SHCs. The SHCs identified here as remote were validated with the program managers. This validation showed that, for many SHCs, porters took non-motorable routes to deliver vaccines. Thus while Google Maps showed the Time-to-Supply greater than 40 min for these SHCs, actual travel times were less. Such SHCs were not considered remote.

### 2.3 Step 2: Realignment of remote SHCs and associated session sites to alternative existing CCPs

The results of the first step of identification of remote SHCs became inputs for the second step. In the second step, a realignment of remote SHCs and their associated session sites to pre-existing alternative CCPs was proposed. Existing CCPs from which the Time-to-Supply to these SHCs was less than 40 min were considered for this realignment. The realignment of remote SHCs and their corresponding session sites to alternative CCPs for resolving the Time-to-Supply challenge was preferred over establishing new CCPs because realignment entailed only an administrative rearrangement with no requirement for capital investment or human resource and infrastructure augmentation.

To further ensure administrative acceptability of the proposed realignment solution, only combinations of SHCs and alternative CCPs situated within the same block of a district were considered. Realignment of an SHC was considered feasible only if it did not decrease the SHC load for an existing CCP below the minimum required load for that CCP based on AVD porter economic analysis. All remaining remote SHCs which could not be realigned to an existing CCP in this step became inputs for the third step.

### 2.4 Step 3: Identification of new CCPs

For the remote SHCs where the Time-to-Supply could not be resolved through realignment to an existing CCP, identification and opening of new CCPs in the block was required. Only HFs which were CHCs and PHCs were considered as candidate locations for new CCPs. While SHCs had the greatest presence and thus formed the likeliest candidate for conversion to new CCPs, they were not ideal candidates due to a variety of resource constraints related to limited human resource availability, electricity supply, mobile and broadband internet connectivity and access to all-weather roads.

The optimization problem of identifying new CCPs from the candidate locations to ensure maximum coverage of remote sites in each block was formulated, as listed in the Appendix A. The objective function of the problem maximized the coverage of demand points, with two kinds of decision variables present— *Y_j_* (which decided if a given candidate location is selected for establishing the facility), and *X_ij_* (which decided which pair of demand points and candidate location was selected). The problem was structured as a Binary Integer Programming problem, as all decision variables could only take “yes” or “no” values. In total, after the realignment in Step 1, 32 blocks (with 98 CCPs and 144 SHCs overall) remained where new CCP locations were to be identified, and thus 32 separate optimization problems were formulated.

To solve the optimization problem, two competing algorithms- greedy adding (which was modelled in Microsoft Excel), and Branch & Bound algorithm (using Excel Solver’s Simplex LP function) were used to first solve a test set of problems, comprising of 34.3% (n = 11) of the overall problem set, and the values of objective function (performance measure) were compared. Greedy algorithms have been used frequently to solve location-allocation problems of various forms [40], [41], [42] previously.

The results of solutions from the two algorithms are compared below. According to Table 2, identical solutions were obtained from using the two algorithms. For the overall problem set, greedy adding was chosen over Branch & Bound as it provides near-optimal solutions (although optimal for this problem set) in polynomial time for certain types of np-hard problems such as the Maximal Coverage Location Problems (MCLP) formulated here, whereas exact algorithms such as Branch & Bound require exponential time. Additionally, setting up the optimization problem by defining all the decision variable and constraints also requires significantly more time for Branch & Bound method as compared to the Greedy Adding algorithm [43].

**Table 2:** Values of performance measure Z for test set of 11 optimization problems.

Dimensions (Decision Variables × Constraints)	Greedy Adding	Branch & Bound
45 × 88	8	8
30 × 63	9	9
6 × 9	1	1
8 × 15	0	0
8 × 15	0	0
9 × 14	2	2
9 × 14	1	1
10 × 25	2	2
12 × 18	1	1
14 × 35	1	1
15 × 28	1	1
30 × 63	2	2

## 3 Results

With an intent to gauge the impact of the network optimization and extension exercise, the metrics related to reduction in number of remote sites, and average reduction in TTS overall, for realigned sites, and for remote sites attached to new CCPs were measured. Additionally, the reduction in SHC load resulting from the new CCP establishment was measured.

The AVDS porter economic analysis provided the basis for the minimum load constraint. In cases where a porter served two SHCs, the net profit was negative for five out of six possible combinations of hard-to reach and easy-to-reach sites across minimum and maximum distance ranges. Similarly, in cases where a porter served three SHCs, the net profit was negative for four out of eight possible combinations. Only when the total number of SHCs delivered to was equal to or greater than four, was the employment profitable for a porter for all combinations of hard-to reach and easy-to-reach sites, and thus the minimum SHC load for a CCP for the purpose of optimization was considered to be four. The results for the economic analysis are shown in Table 3.

**Table 3:** AVDS porter economic analysis.

Total Sessions			Distance Range		Net Profit over Opportunity Costs	
*Hard-To-Reach*	*Easy-To-Reach*	*Total*	*Min (Km)*	*Max (Km)*	*Min*	*Max*
2	0	2	14	20	₹−133.31	₹−140.19
1	1	2	64	70	₹−100.50	₹−113.94
0	2	2	68	80	₹2.94	₹−17.38

3	0	3	18	30	₹−49.88	₹−63.63
2	1	3	64	70	₹−10.50	₹−23.94
1	2	3	68	80	₹92.94	₹72.63
0	3	3	72	90	₹196.38	₹169.19

4	0	4	22	40	₹33.56	₹12.94
3	1	4	64	70	₹79.50	₹66.06
2	2	4	68	80	₹182.94	₹162.63
1	3	4	72	90	₹286.38	₹259.19
0	4	4	76	100	₹389.81	₹355.75

Table 4 provides the summary results from Steps 1, 2, and 3. In step 1, from the overall list of 1464 SHCs in 53 blocks of eight districts, 274 SHCs across 40 blocks were identified as remote, based on the travel times sourced from the Google Maps Distance Matrix API. The results obtained here were validated with the program managers, and based on their inputs, 42 SHCs were reclassified as non-remote. This was due to porters following paths that were classified as non-motorable by Google Maps.

**Table 4:** Summary results.

Total SHCs	1500
Step 1: Identification of remote SHCs	
Total Remote SHCs	232
Average TTS (Overall)	26.50 min

Step 2: Realignment of remote SHCs to existing CCPs	
Realigned SHCs	73
Average TTS (Realigned SHCs, current)	55.80 min
Average TTS (Realigned SHCs, post realignment)	23.57 min

Step 3: Identification of new CCPs	
New CCPs identified	22
SHCs attached to new CCPs	59
Average TTS (SHCs attached to new CCPs, current)	56.95 min
Average TTS (Post attachment to new CCPs)	23.07 min

Total Remote SHCs (after Steps 1, 2, 3)	100

In step 2, results from phase 1 were used to identify opportunities for realignment of remote SHCs to existing CCPs in the same block. In all, 88 SHCs from the 232 remote SHCs could be potentially realigned to a closer CCP. However, for currently mapped CCPs of 15 such SHCs, the SHC load was falling below four, and thus these SHCs were not recommended for realignment. In total, 73 SHCs out of the shortlisted 88 were finally recommended to be realigned. For the CCPs to which these SHCs were realigned, the SHC load rose by 4.29 SHCs on average (from a base of 14.52 SHCs on average, an increase of 29.55%).

**Fig. 2:**
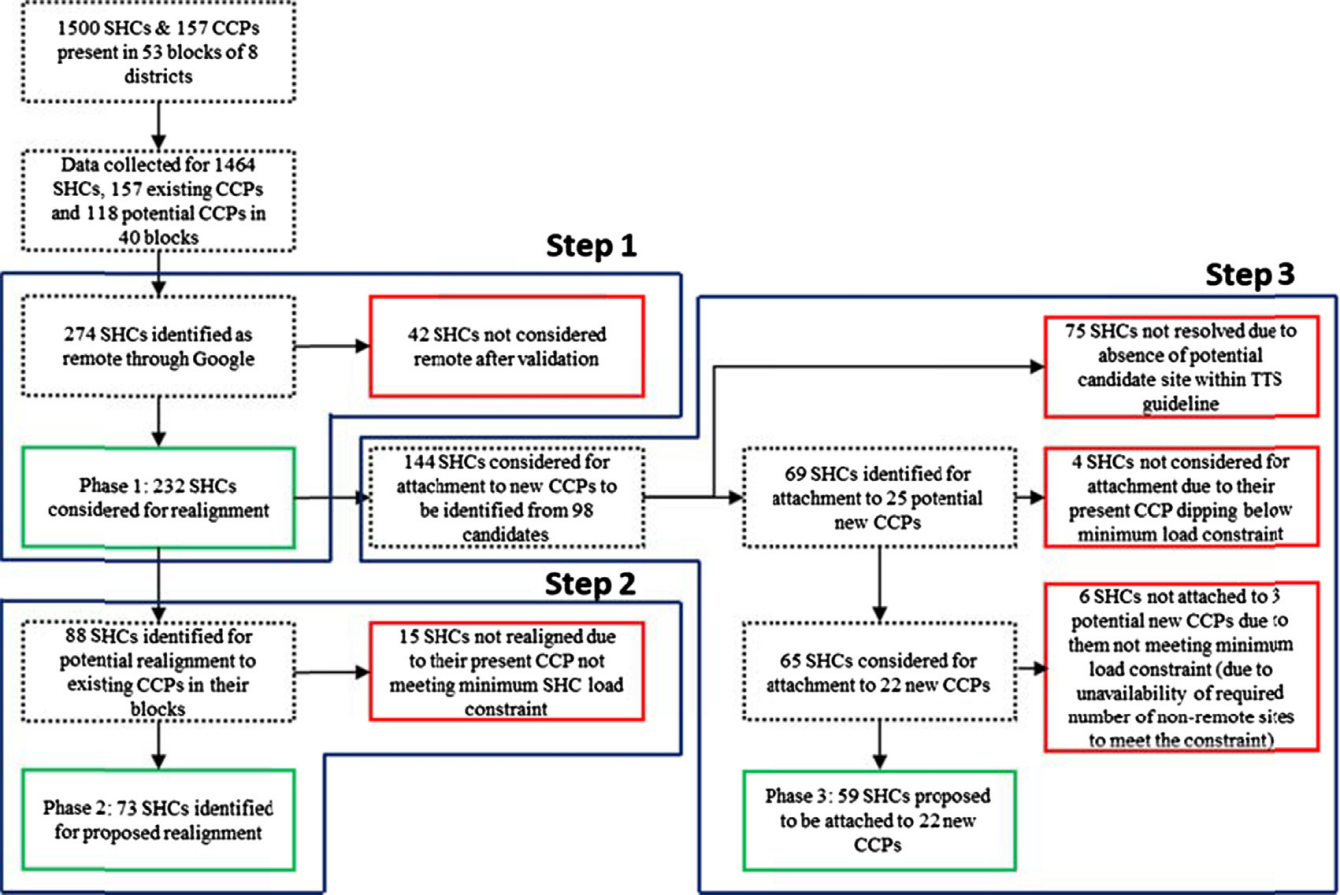
Results of network optimization and extension in 8 districts of Madhya Pradesh.

For the step 3 analysis, the list of remote sites was updated to reflect the realigned SHCs. New CCPs were identified from the given set of CHCs and PHCs in each block. Twenty-five potential new CCPs were identified, of which six were able to serve equal to or more than the minimum load of four SHCs. For the remaining 19 CCPs, additional non-remote sites were identified from within the block for realignment to meet the minimum required SHC load. For three CCPs, a sufficient number of non-remote sites was not present, and thus finally 22 new CCPs were recommended for being setup, of which six served 37 remote SHCs, and the other 16 served 22 remote SHCs, and 42 realigned non-remote SHCs. These 22 new CCPs would thus serve 59 out of the 159 remote SHCs that remained after phase 2. Fig. 2 and Table 5 show the results of phases 1, 2 and 3 for all eight districts. The average overall Time-to-Supply reduction was 13.14 %, and the average Time-to-Supply reduction for remote SHCs realigned to existing CCPs was 57.77 percent. For remote SHCs mapped to new CCPs, the reduction was 59.49 percent on average. Table 6 shows the Time-to-Supply reduction on average in each block, for the overall case, realigned SHCs, and SHCs attached to new CCPs respectively.

**Table 5:** District wise results of steps 1, 2, and 3.

District	Block	Existing CCPs	Candidate CCPs	No. of SHCs	Remote SHCs (Phase 1)	Realigned SHCs (Phase 2)	Remote SHCs attached to new CCPs (Phase 3)
D1	D1B1	6	0	43	0	0	0
	D1B2	3	0	31	15	9	0
	D1B3	3	2	36	11	8	0
	D1B4	4	0	31	3	0	0
	D1B5	6	1	50	0	0	0
	D1B6	9	1	68	14	9	0
	D1B7	5	2	38	0	0	0
D2	D2B1	5	3	24	3	3	0
	D2B2	3	2	27	4	1	0
	D2B3	3	3	21	1	0	0
	D2B4	2	1	13	0	0	0
	D2B5	0	1	21	0	0	0
	D2B6	2	1	7	1	0	0
	D2B7	2	2	26	6	0	1
	D2B8	5	3	29	6	0	0
	D2B9	3	7	34	2	0	1
D3	D3B1	1	0	20	0	0	0
	D3B2	3	3	30	9	0	9
	D3B3	3	1	23	12	8	0
	D3B4	2	1	31	3	1	1
	D3B5	2	0	19	4	1	0
	D3B6	2	4	26	1	0	1
	D3B7	1	1	26	2	0	0
D4	D4B1	1	2	15	0	0	0
	D4B2	2	1	22	11	0	4
	D4B3	3	2	32	3	0	1
	D4B4	5	2	22	5	1	2
	D4B5	4	2	28	4	1	0
D5	D5B1	1	0	8	0	0	0
	D5B2	2	2	23	0	0	0
	D5B3	1	0	16	2	0	0
	D5B4	4	0	51	5	0	0
	D5B5	2	4	21	3	1	1
	D5B6	5	6	37	2	0	2
	D5B7	5	6	37	0	0	0
D6	D6B1	8	1	75	9	4	0
	D6B2	3	6	66	12	4	5
	D6B3	4	3	58	20	11	2
D7	D7B1	1	0	8	0	0	0
	D7B2	1	4	20	2	0	2
	D7B3	4	1	16	2	0	0
	D7B4	2	4	19	0	0	0
	D7B5	1	1	18	0	0	0
	D7B6	2	3	20	2	0	2
	D7B7	1	3	25	2	0	1
	D7B8	1	0	4	3	0	0
	D7B9	2	5	17	13	0	8
D8	D8B1	3	4	32	8	4	4
	D8B2	2	4	24	1	0	0
	D8B3	2	2	24	1	0	1
	D8B4	4	5	37	7	5	1
	D8B5	2	4	27	11	0	10
	D8B6	4	2	24	7	2	0
**Total**		**157**	**118**	**1464**	**232**	**73**	**59**

**Table 6:** Time-to-Supply reduction due to realignment and new CCP establishment.

District	Block	Average % reduction of Overall TTS	Average % reduction of TTS for realigned SHCs	Average % reduction of TTS for SHCs attached to new CCPs
D1	D1B1	–	–	–
	D1B2	30.07%	72.37%	–
	D1B3	43.24%	92.14%	–
	D1B4	–	–	–
	D1B5	–	–	–
	D1B6	22.77%	66.71%	–
	D1B7	–	–	–

D2	D2B1	11.40%	46.71%	–
	D2B2	3.22%	40.63%	–
	D2B3	–	–	–
	D2B4	–	–	–
	D2B5	–	–	–
	D2B6	–	–	–
	D2B7	3.84%	–	41.61%
	D2B8	–	–	–
	D2B9	4.85%	–	52.07%

D3	D3B1	–	–	–
	D3B2	27.31%	–	53.94%
	D3B3	26.62%	59.26%	–
	D3B4	11.94%	47.03%	36.82%
	D3B5	3.15%	31.23%	–
	D3B6	14.54%	–	99.62%
	D3B7	–	–	–

D4	D4B1	–	–	–
	D4B2	26.88%	–	78.13%
	D4B3	3.95%	–	19.01%
	D4B4	15.38%	41.98%	76.98%
	D4B5	3.51%	54.43%	–

D5	D5B1	–	–	–
	D5B2	–	–	–
	D5B3	–	–	–
	D5B4	–	–	–
	D5B5	16.46%	88.46%	28.67%
	D5B6	12.44%	–	78.26%
	D5B7	–	–	–

D6	D6B1	7.66%	68.16%	–
	D6B2	25.39%	48.26%	82.56%
	D6B3	20.33%	65.07%	38.32%

D7	D7B1	–	–	–
	D7B2	15.77%	–	60.53%
	D7B3	–	–	–
	D7B4	–	–	–
	D7B5	–	–	–
	D7B6	17.87%	–	47.93%
	D7B7	11.54%	–	63.41%
	D7B8	–	–	–
	D7B9	41.78%	–	73.44%

D8	D8B1	18.92%	39.70%	53.05%
	D8B2	–	–	–
	D8B3	10.40%	–	32.25%
	D8B4	10.72%	33.86%	26.97%
	D8B5	35.65%	–	57.39%
	D8B6	12.82%	61.80%	–
**Total**		**13.14%**	**57.77%**	**59.49%**

Thus, considering the results of phase 2 and 3 together, 56.89 percent (132 from 232) of the remote SHCs would now be attached to a CCP less than 40 min away. In all, 100 remote SHCs could not be covered by establishing new CCPs, which was due to either the absence of any PHC or CHC within 40 min of travel time (for 75 sites) or due to the existing or potential new CCP not meeting the minimum requirement of four SHCs (for 25 sites).

**Fig. 3:**
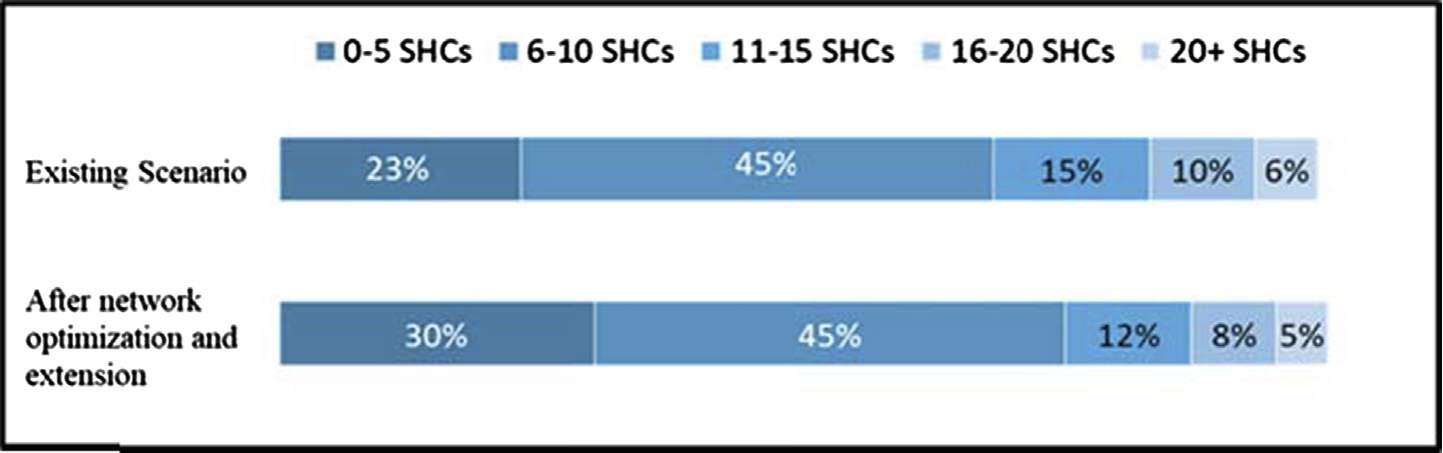
SHC load distribution for CCPs.

**Fig. 4:**
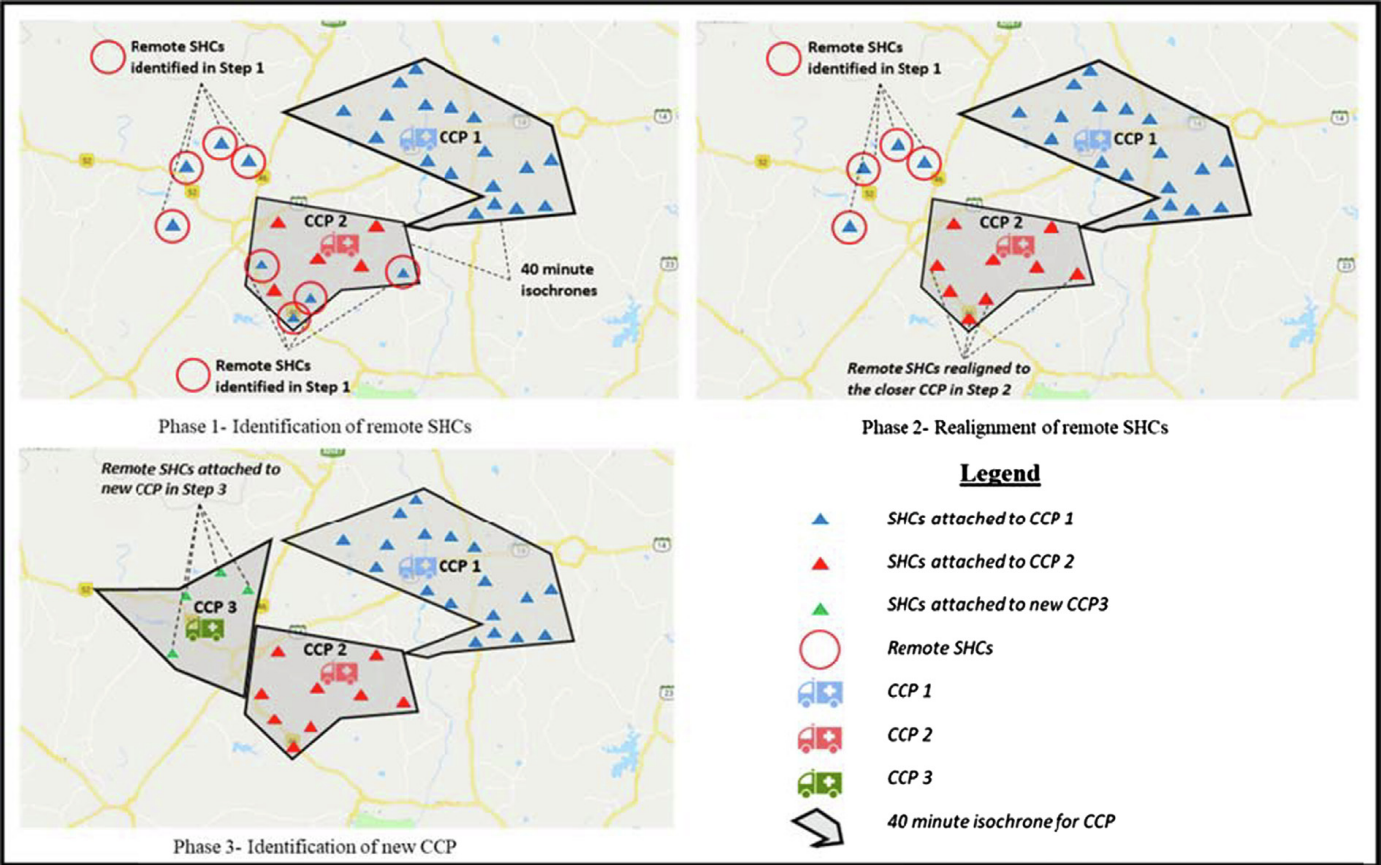
Results of network optimization and extension in an urban block of a northern district.

The average SHC load on CCPs decreased from 9.76 SHCs per CCP to 9.08 SHCs per CCP, due to the establishment of new CCPs, a decrease of 6.97 percent. Fig. 3 shows the SHC load distribution for CCPs, before and after network optimization and extension. Fig. 4 shows the results of this research for a representative urban block in a northern district, where four SHCs were reshuffled, and four were attached to a new proposed CCP.

## 4 Discussion and limitations

To ensure availability of vaccines at session sites, improvements are required in vaccine inventory management, timeliness of deliveries, and HCW capacity, among other factors. Vaccine potency is impacted by freezing and heat exposures during transportation which in turn may become less likely through reduction in travel duration outside of active Cold Chain. Increasing the number of distribution centers reduces reserve stock levels and inventory-holding costs while providing reliable and on-time delivery of vaccines to session sites [44]. It also decreases the duration of transportation and since most exposures to freezing during last mile transport occur due to poorly conditioned ice packs, reduced transportation duration may contribute to better conditioning as Cold Chain Handlers would have more time available to follow appropriate conditioning processes and guidelines. In the extreme weather conditions of central India, reduced travel times may also help ensure that vaccines remain in the safe temperature zone until the open vials are returned to CCPs post immunization sessions. Session start timings may be positively impacted due to on-time availability of vaccines through AVDS porters, however this would remain a function of timeliness of both the porters and the HCWs.

Developing countries operate under resource constraints and therefore while extending the last tier of distribution network opportunities for optimization must be identified and acted upon. The realignment results identified in the paper help to optimize the supply chain by decreasing the travel times and distances between CCPs and session sites, while incurring minimal costs, as they involve only an administrative rearrangement and redesign of delivery and schedules at the local level for the most part.

Optimal decision making around facility location is essential, since the expansion of the iSC network is a binding long-term decision, involving both capital expenditures (such as equipment, building setup, civil and electrical work) and operating costs (related to transportation, electricity, human resources, and administration) [45]. Operating costs by their nature are recurrent and thus any savings ensured through optimal locations would accrue over the life of the optimised system.

Under the National Immunization Schedule of UIP, the birth dose for Hepatitis-B vaccine should be administered within 24 h of birth. Since CHCs and PHCs, where childbirths are facilitated by the ANMs, may not always have vaccines available at onsite during non-session days, ensuring birth dose availability and administration can prove a challenge. At health facilities now converted to CCPs, all vaccines under India’s UIP would be available, and thus availability of the birth dose vaccine for Hepatitis-B can be ensured, leading to increased coverage.

Both realignment and new CCP establishment could lead to reduced local delivery costs, due to reclassification of SHCs from hard-to-reach to easy-to-reach. Since the porters are compensated on the basis of number of trips by category, receiving USD 2.88 for hard-to-reach areas and USD 1.30 for easy-to-reach areas, the savings from such a reclassification may exceed 50 percent in some cases. Additionally, new CCP creation could lead to a more equitable distribution of SHC load and may lower the overall burden on the healthcare workers at sites where the SHC load has decreased. Equity is one of the strategic objectives laid out in both the national MoHFW’s cMYP (2018–22) and the WHO’s Global Vaccine Action Plan (2011–2020) [46]. By ensuring that potent vaccines become available in a timely manner to the beneficiaries, the work described here may contribute to this objective.

As in other studies, the model of the supply chain approximates the real-world scenario. First, SHCs have been used as proxy for session sites and the optimization has accounted for the average travel time from SHCs to session sites. While this assumption may hold valid in many cases, the session sites falling beyond the average travel time may not be resolved. For such sites and other remote sites remaining unresolved after the new CCP identification process alternative methods such as provision of passive cold storage devices or mobile strategy for outreach may be explored.

Second, while the Greedy Adding algorithm deployed here provided optimal solutions in this problem instance, using exact algorithms may prove to be more effective for problem instances where constraints are fewer, and runtime or storage challenges are not present. Greedy Adding prioritizes locally optimal choices at each stage to reach global maxima, trading off exactness with ease of formulation and application, and thus may not be suitable to certain problem instances with fewer dimensions.

Third, while the focus has been on reducing the direct travel times to SHCs from CCPs, if during one trip the AVDS porters deliver vaccines to more than one session site, the proposed network optimization could be augmented by a route-planning approach. Such an approach would require geo-coordinates of session sites, and thus may form a future course of research.

Fourth, due to remoteness of geographies and limited mobile network connectivity, there may be inaccuracy in capturing location information for sites. Further, travel times and distances have been sourced from Google Maps and the accuracy of this data may vary by seasons (when certain routes may close due to rains and flooding) or due to incomplete or inaccurate maps. As new roads are built, one can expect distances and travel times to reduce further and thus while the approach would stay valid more optimal locations for new CCPs may be identified. In order to ensure that the proposed selection is implementable, consultations with local staff are critical. While the information on remoteness was validated with program managers, the organizational and local level knowledge present with such managers may not be sustainable due to employee attrition or transfers.

Fifth, the approach and findings hold validity for Routine Immunization and not for mass immunization campaigns as the block-boundary constraints may be relaxed for immunization campaigns.

Sixth, a constraint on the maximum number of SHCs that could sustainably be attached to a new CCP and the closely related problem of overburdening of CCPs due to addition of remote SHCs in case of realignment have not been considered.

Finally, the larger impact of the approach on the potency of vaccines, timeliness of sessions, and beneficiary trust in the public health system and consequently the Full Immunization Coverage remains to be seen.

## 5 Conclusion

As per the authors’ knowledge, the work described here is the first formal approach for optimization and location selection of the last tier of the immunization supply chain with a focus on increasing equity for the beneficiaries. The key contribution of this paper is to develop a practical approach for optimization of the last tier of immunization supply chain, potentially impacting vaccine quality, ensuring timeliness of sessions, and reducing burden on HCWs, among other less tangible spill over benefits. Based on prior work that the authors have done in different states, they feel that the approach laid out here is replicable across other countries, and potentially useful to governments or partner organizations looking to identify optimization opportunities in the immunization supply chain, or looking to expand its last tier.

## 6 ICJME statement

All authors attest that they meet the ICMJE criteria for authorship.

## Funding

This work was funded by a grant from the Bill & Melinda Gates Foundation – United States. The views expressed herein are solely those of the authors and do not necessarily reflect the views of the Foundation.

## CRediT authorship contribution statement

**Varun Srivastava:** Conceptualization, Formal analysis, Methodology, Writing – original draft, Visualization. **Manish Ratna:** Conceptualization, Methodology, Software, Investigation, Data curation, Writing – original draft, Visualization. **Arindam Ray:** Conceptualization, Methodology, Validation, Writing – review & editing, Funding acquisition. **Santosh Shukla:** Conceptualization, Methodology, Validation, Writing – review & editing, Supervision. **Vipin Shrivastava:** Conceptualization, Methodology, Writing – review & editing, Supervision. **Nitin Kothari:** Conceptualization, Methodology, Validation, Investigation, Data curation, Supervision, Writing – review & editing, Project administration. **Amandeep Gupta:** Conceptualization, Software, Formal analysis, Investigation, Data curation, Writing – review & editing. **Meenal Kukreja:** Conceptualization, Methodology, Validation, Writing – review & editing, Supervision, Project administration. **Harkabir Singh Jandu:** Conceptualization, Methodology, Validation, Writing – review & editing, Supervision, Project administration.

## Declaration of Competing Interest

The authors declare the following financial interests/personal relationships which may be considered as potential competing interests: Dr Santosh Shukla and Er Vipin Shrivastava work for the Directorate of Health Services, Government of Madhya Pradesh. Amandeep Gupta, Harkabir Singh Jandu, Manish Ratna, Meenal Kukreja, Nitin Kothari, and Varun Srivastava work for the Clinton Health Access Initiative (CHAI), which was the implementation partner for the research. Dr Arindam Ray works for the Bill & Melinda Gates Foundation (BMGF), which provided the financial support to CHAI during this research. The views expressed in this article are those of the authors and do not necessarily represent the views, decisions, or policies of the institutions with which they are affiliated.

## Appendix A

### A.1 Problem formulation

Sets
*I*: The set of demand points (remote SHCs)
*J*: The set of candidate locations (CHCs and PHCs currently not a CCP)
*N_j_*: The set of all candidate locations which can cover demand point within the time constraint
*i ∈ I* (*N_j_=*{ *j ∈ J: d_ij_ ≤ D*})
Input Parameters:
*d_ij_*: The travel time from demand point *i ∈ I* to candidate location *j ∈ J*
*D*: The maximum acceptable one-way travel time between one pair of candidate location and demand point (in this case, *D* = 40 min)
*p*: The maximum number of candidate locations to be established (in this case, *p* = [*J* ])
Decision Variables:
*X_ij_*: 1, if demand point *i* is allocated to candidate location *j* ; 0 otherwise
*Y_j_*: 1, if facility is established at candidate location *j* ; 0 otherwise
Formulation:

(1) $$\max\Sigma_{i\in I}\Sigma_{j\in Nj}X_{\mathrm{ij}}$$

subject to

(2) $$Y_{j}-\Sigma_{i\in I}X_{\mathrm{ij}}\geq 0$$

(3) $${\Sigma_{j}}_{\in J}X_{\mathrm{ij}}\leq 1\forall i\in I$$

(4) $$d_{\mathrm{ij}}X_{\mathrm{ij}}\leq D\forall i\in I,j\in J$$

(5) $$X_{\mathrm{ij}}\in \left\{0,1\right\}\forall i\in I,j\in J$$

(6) $$Y_{j}\in \left\{0,1\right\}\forall ,j\in J$$

In this formulation, the objective function Eq. (1) maximizes the coverage of demand points. Constraint Eq. (2) ensures demand points are covered only by established candidate locations. Constraint Eq. (3) ensures that no demand point is covered by more than one candidate location. Constraint Eq. (4) limits solutions to pairs where one-way travel time is lower than the maximum permissible travel time *D* = 40 min.

Constraints Eqs. (5), (6) are integrality constraints, ensuring that *X_ij_* and *Y_j_* take only binary values.

### A.2 Description of Greedy Adding algorithm

In each iteration, the Greedy Adding algorithm identified one candidate location for establishing a new CCP and identified demand locations to be covered by that candidate location. This was done by selecting the candidate location that could serve the maximum number of demand points given the constraints. If two CCPs were able to serve the same number of demand points, then the CCP for which sum of total travel time was minimum was chosen. In step two, the set of candidate locations and demand points was updated to exclude the previously established facilities and covered demand points. The algorithm continued until no more facilities could be established while still meeting the constraints or till all possible candidate locations were selected for establishment. For the identified potential new CCPs where the minimum load of SHCs was not being satisfied by remote SHCs, additional non-remote sites from surrounding areas in the block were identified for realignment to the new CCP. In case the required number of non-remote SHCs were not available for realignment, or such a realignment decreased the SHC load at their existing CCPs below the minimum, the potential new CCP was not considered feasible. Priority of realignment was given to non-remote SHCs for which decrease in Time-to-Supply was maximum.
